# LONG-TERM EXPOSURE TO FINE PARTICULATE MATTER AND DEPRESSIVE SYMPTOMS AMONG CHINESE OLDER ADULTS

**DOI:** 10.1093/geroni/igad104.2679

**Published:** 2023-12-21

## Abstract

There is a growing body of evidence suggesting that air pollution, especially PM2.5 (particulate matter with a diameter of 2.5 micrometers or less), has been associated with adverse mental health outcomes, but the long-term association with depressive symptoms remains unclear. The purpose of this study is to examine the long-term effect of cumulative exposure to PM2.5 on depressive symptoms among Chinese older adults. Using a nationally representative sample of older adults in China (N=16,064), we tracked their depressive symptoms over a 16-year period (2002-2018). Air pollution data were linked to respondents using provincial identifiers. Three-level random intercept linear regression models that account for the nesting of repeated observations (level-1) within individuals (level-2) and individuals within provinces (level-3) were used to examine the long-term cumulative effects of PM2.5 exposure on depressive symptoms. We found that every 10-μg/m3 increase in cumulative exposure to PM2.5 increased the levels of depressive symptoms by 0.28 points (β = .28, p < .01), controlling for individual-level sociodemographic characteristics and social support, health-related behaviors, and health status. Furthermore, the association between cumulative PM2.5 exposure and more depressive symptoms is more pronounced for urban residents than for rural residents. Our analysis shows that air pollution is a risk factor for depression in older Chinese adults. Findings suggest that stronger PM2.5 regulations, especially in urban areas, may enhance mental health.

